# Silicon-based optoelectronics: progress towards large scale optoelectronic integration and applications

**DOI:** 10.1007/s12200-022-00030-7

**Published:** 2022-05-31

**Authors:** Dingshan Gao, Zhiping Zhou

**Affiliations:** 1 Wuhan National Laboratory for Optoelectronics, Huazhong University of Science and Technology, Wuhan, 430074 China; 2 State Key Laboratory of Advanced Optical Communication Systems and Networks, School of Electronics, Peking University, Beijing, 100871 China

In the past half century, silicon-based microelectronics and optical fiber communication have triggered a far-reaching information technology revolution, which has moved human society into a high-speed information age. The demand for communication capacity and speed is growing exponentially. On the other hand, data center and high-performance computing are facing bottlenecks of speed, bandwidth, and energy consumption of electrical interconnections. Silicon-based optoelectronics has become the key technology to break through these bottlenecks. Thanks to the advantages of high refractive index, capable in small active components, and CMOS compatible process, silicon can achieve a large-scale optoelectronic integration on a micro-chip with low cost and low energy consumption. This has become a hot alternative for the chip industry. In addition, silicon-based optoelectronics has enabled a series of new study fields such as mid-infrared communication, microwave optoelectronics, lab-on-chip, quantum communication, optoelectronic computing, and chip scale lidar.

This special issue on “Recent Advances in Silicon Photonics” encompasses the recent developments in the devices and applications in the field. With five review papers and four original research articles included, this special issue focuses on key devices and their applications in data center coherent interconnections, optoelectronic computing, integrated quantum circuit, and silicon-based optoelectronic hybrid integration.

Liu et al. [1] reviewed the state-of-art of thermo-optic phase shifters on SOI substrate. Seiler et al. [2] evaluated and compared the performances of silicon-based optoelectronic components for coherent O-band data center interconnections. Qiu et al. [3] summarized the developing trend of integrated optical directed logic operations in which the operands are electrons, and the operation results are photons. Adcock et al. [4] explored the prospects of dynamic quantum circuits on hybrid thin-film lithium niobate on silicon (TFLN/Si) photonics. Tan et al. [5] identified the key building blocks for the integrated circuits for electronics-photonics convergence and reviewed their recent advances. Cheng et al. [6] proposed a systematic solution to extend the matrix computation of micro-ring array from real-valued field to complex-valued field, and from small scale to large scale matrix computation. Georgieva et al. [7] reported polarization combining 2D grating couplers (2D GCs) on amorphous Si:H, fabricated in the backend of a line of a photonic BiCMOS platform. Tao et al. [8] demonstrated an ultra-compact, low-loss, slot-strip converter with polarization insensitivity based on the multimode interference effect. Ma et al. [9] introduced a method to obtain complete photonic bandgap (CPBG) in a silicon nitride (Si_*x*_N_*y*_) photonic crystal slab.

The nine papers in this special issue comprise only the partial progress in the field of silicon-based optoelectronics. We believe that this special issue will provide the readers with the latest research trends in this field and around their strong interest in this subject matter, further stimulating more cutting-edge research in the future.
